# Solubilization of Charged Porphyrins in Interpolyelectrolyte Complexes: A Computer Study

**DOI:** 10.3390/polym13040502

**Published:** 2021-02-06

**Authors:** Karel Šindelka, Zuzana Limpouchová, Karel Procházka

**Affiliations:** 1 Department of Molecular and Mesoscopic Modelling, Czech Academy of Sciences, Institute of Chemical Process Fundamentals, Rozvojová 1, 165 02 Prague, Czech Republic; sindelka@icpf.cas.cz; 2 Department of Physical Chemistry, Faculty of Science, Charles University, Hlavova 8, 128 00 Prague, Czech Republic; zuzana.limpouchova@natur.cuni.cz

**Keywords:** computer simulations, porphyrin, electrostatic co-assembly, IPEC, solubilization

## Abstract

Using coarse-grained dissipative particle dynamics (DPD) with explicit electrostatics, we performed (i) an extensive series of simulations of the electrostatic co-assembly of asymmetric oppositely charged copolymers composed of one (either positively or negatively charged) polyelectrolyte (PE) block A and one water-soluble block B and (ii) studied the solubilization of positively charged porphyrin derivatives (P${}^{+}$) in the interpolyelectrolyte complex (IPEC) cores of co-assembled nanoparticles. We studied the stoichiometric mixtures of 137 A${}_{10}^{+}$B${}_{25}$ and 137 A${}_{10}^{-}$B${}_{25}$ chains with moderately hydrophobic A blocks (DPD interaction parameter $a_{AS}=35$) and hydrophilic B blocks ($a_{BS}=25$) with 10 to 120 P${}^{+}$ added ($a_{\mathrm{PS}}=39$). The P${}^{+}$ interactions with other components were set to match literature information on their limited solubility and aggregation behavior. The study shows that the moderately soluble P${}^{+}$ molecules easily solubilize in IPEC cores, where they partly replace PE${}^{+}$ and electrostatically crosslink PE${}^{-}$ blocks. As the large P${}^{+}$ rings are apt to aggregate, P${}^{+}$ molecules aggregate in IPEC cores. The aggregation, which starts at very low loadings, is promoted by increasing the number of P${}^{+}$ in the mixture. The positively charged copolymers repelled from the central part of IPEC core partially concentrate at the core-shell interface and partially escape into bulk solvent depending on the amount of P${}^{+}$ in the mixture and on their association number, $A_{S}$. If $A_{S}$ is lower than the ensemble average ${\langle A_{S}\rangle }_{n}$, the copolymer chains released from IPEC preferentially concentrate at the core-shell interface, thus increasing $A_{S}$, which approaches ${\langle A_{S}\rangle }_{n}$. If $A_{S}>{\langle A_{S}\rangle }_{n}$, they escape into the bulk solvent.

## 1. Introduction

Porphyrins, matalloporphyrins, and various porphyrin-based complex molecules belong to the most ubiquitous heterocyclic compounds found in nature. They play an important role in a number of processes vital for sustaining life on Earth, e.g., in photosynthesis, the transport of oxygen in living organisms and parts of enzymes and vitamins [1]. Synthetic porphyrins and metalloporphyrins exhibit similar biological and physicochemical properties as their natural analogues and offer a number of important applications in medicine (treatment of diseases [1,2,3,4,5], biological sensing and imaging [1,6,7], and drug-delivery and photochemical internalization systems [8]), chemistry (chemical analysis [9] and photocatalysis [10]), physics (nonlinear optics [11] and photovoltaics [12]), and the industry [13].

The most common application in medicine (photodynamic therapy [3,14]) as well as other applications assumes the interaction of excited porphyrin molecules with other compounds (oxygen, DNA, etc.). The photophysics of porphyrins is rich [15,16]; their absorption spectra consist of an intense and fairly broad Soret band (S${}_{0}\to$ S${}_{2}$ transition) with maximum absorption slightly above 400 nm and of multiple Q-bands (S${}_{0}\to$ S${}_{1}$ transitions) in the region 500 to 650 nm. The Q-bands are appreciably weaker (more than ten times) than the Soret band, but they are important for most applications because near-infrared light can penetrate deeper into biological tissues than the short-wavelength violet light. The position and intensity of Q-bands is influenced by association. The fluorescent behavior is even more complex. When exited below 400 nm, porphyrins exhibit S${}_{2}\to$ S${}_{0}$ (relatively weak) emission in the region 400–550 nm and, thus, violate the Kasha rule [17]. Their S${}_{1}\to$ S${}_{0}$ emission in the region 550–800 nm is strongly affected by the ground-state association due to π–π interaction of large rigid aromatic rings. The principles of formation of weakly bound dimers and higher aggregates, and their fluorescent properties were studied and explained by Kasha in 1965 [18]. He classified the aggregates according to the angle θ between the absorption transition dipole moment and the line connecting the gravity centers of monomers. Two extreme cases are (i) the “head-to-tail” J-dimers/aggregates, in which the transition moment is parallel to the connecting line ($\theta =0^{\circ }$) and (ii) coplanar “face-to-face” H-dimers/aggregates when $\theta =90^{\circ }$. According to theory, extreme J-aggregates are not fluorescent and their absorption spectra are blue-shifted with respect to the spectra of a monomer. The absorption of J-aggregates is red-shifted, and their red-shifted emission is strong. As the aggregates are only weakly bound, it is not surprising that the association process, the structure of aggregates, and the monomer-to-aggregate equilibrium are sensitively influenced by interaction with the microenvironment. In reality, the arrangement of monomers in aggregates varies and depends on external conditions. The range of possible angles is wide ($0<\theta <90^{\circ }$). Strongly fluorescent species with red-shifted spectroscopic characteristics and with $0<\theta <54.70^{\circ }$ ($\theta_{M}=54.70$ is the magic angle) are classified as J-aggregates, and weakly fluorescent species with blue-shifted characteristics, the structure of which corresponds to $55.40<\theta <90^{\circ }$, are classified as H-aggregates. Thorough experimental and theoretical studies of porphyrin, photophysics revealed that the highly symmetric D${}_{4h}$ aromatic rings form four types of structurally different aggregates: two J- and two H-dimers, which form under different conditions and can be experimentally discerned on the basis of their photophysical properties [15,16,19,20].

Medical applications assume the administration of porphyrins into aqueous media of living organisms. The large aromatic ring is strongly hydrophobic and apt to stack π–π, which results in low water-solubility and significant aggregation. Any efficient medical treatment requires specific interactions of certain forms of excited porphyrins with biologically important compounds. Therefore, the main task and real challenge for researchers is the efficient targeted delivery of selected porphyrin forms. Up until now, several different strategies have been designed, tested, and successfully applied. As the main target of porphyrin action is DNA, one of the promising possibilities consists in attaching porphyrin and a certain (purposely designed) quencher to opposite ends of a short single-strain DNA motif [21]. Short DNA improves the solubility of the construct. It forms a loop, and the porphyrin emission is quenched. After the attachment of the porphyrin–DNA construct to native DNA, the loop opens and the porphyrin photo-activity is restored. Another possibility of fluorescence reactivation consists in enzymatic cleavage of the quencher [22]. The most common approach aimed at improving porphyrin solubility in water is the synthesis of positively or negatively charged di-, tri-, or tetra-substituted derivatives, e.g., tetratolylporphyrin substituted by pyridinium groups [23,24]. Even though the positive charge is preferable because it secures the attractive interaction with negatively charged DNA, the negatively charged 5,10,15,20-tetrakis-(4-sulfonatophenyl)-porphyrin (TPPS) has been also synthesized and amply studied [1,2,25]. Recently, various porphyrin-containing systems have been designed and tested [26,27,28,29]. Gröhn and coworkers studied the electrostatic association of porphyrins with oppositely charged polyelectrolytes and described several different structures depending on the chain architecture and stiffness [30,31]. An interesting paper was published by Zhao et al. [25]. The authors studied the co-micellization of poly(4-vinyl pyridine)-*b*-poly(ethylene oxide) (P4VP-PEO) and poly(2-(dimethylamino)ethyl methacrylate)-*b*-poly(N-isopropylacrylamide) (PDMAEMA-PNIPAM) with TPPS in aqueous media and found that the hydrophobic π–π interaction of pyridine cycles with porphyrin plays an important role and contributes to the formation of associates at elevated pHs when PE chains are not ionized.

In recent decades, core-shell nanoparticles prepared either by self-assembly of amphiphilic block copolymers or by co-assembly of polyelectrolytes, thermodynamics of their formation, and their structure and properties have been amply studied by experimentalists and theoreticians, mainly with respect to their promising use in medicine as vessels for targeted drug delivery [32,33,34,35,36,37,38,39,40,41,42,43,44,45,46,47,48,49]. Notably, nanoparticles with insoluble interpolyelectrolyte complex (IPEC) cores stabilized by strongly hydrated shell formed by a neutral water-soluble polymer, e.g., by biocompatible poly(ethylene oxide), represent suitable candidates for transport of ionic drugs. However, to the best of our knowledge, the possibility to incorporate charged porphyrins into IPEC cores of such nanoparticles has not yet been investigated—either experimentally or by simulations. The question if, to what extent, and in which form the large disk-like molecules charged at their outermost periphery would be solubilized in compact IPEC is interesting, and the answer is not a priori obvious for the following reasons: Studies of electrostatic co-assembly of PEs [41,49,50,51,52,53,54,55,56] show that the formation of core-shell associates with segregated domains requires not only the presence of opposite charges on PE blocks (natural prerequisite) but also moderate hydrophobicity of PE backbones and their partial incompatibility with the shell-forming block. From this point of view, 4- or 2-poly(vinyl pyridines) are good candidates because they are insoluble in their non-ionized form in neutral or alkaline solutions and readily soluble at low pH when they are protonated and strongly charged. Hence, the conditions necessary for the formation of IPEC domains suitable for incorporation of porphyrins seem to be well met. However, the main driving force for the formation of nanoparticles with IPEC cores derives neither from electrostatics nor from other interactions but from a considerable increase in entropy upon the liberation of small and mobile counterions, which must stay close to PE chains to compensate for the PE charge and which, upon the formation of electrostatic associates, can escape into bulk solution. Charged porphyrins are relatively large molecules and contain conjugated hydrophobic rings which interact conveniently with polyelectrolytes bearing aromatic rings as pendant groups (e.g., P4PV [25]). They are multiply charged and can serve as electrostatic crosslinking agents, and their incorporation into IPEC liberates small ions. However, they are relatively soluble in aqueous buffers and their molar mass is significantly smaller than that of common PE chains. Therefore, we expect that their partitioning between IPEC and bulk will be a result of intricate enthalpy-to-entropy interplay and will be affected by many factors (chemical nature, stiffness and length of PE blocks, ionic strength of the solution, etc.).

The motivation and aim of the study can be outlined as follows: In up-to-date chemical, biochemical, and biological research, various computer simulations have been successfully used to obtain data difficult to access experimentally or as a replacement of the expensive experimental work [50,57,58,59,60] Therefore, we believe that a computer study based on a model that (i) reflects the planar structure of porphyrins, hydrophobicity of their large aromatic ring, and their tendency to form aggregates; (ii) describes well all non-electrostatic interactions (i.e., not only the interactions between P4PV rings and their interactions with PE chains but also all non-electrostatic interactions between PE blocks and those with the solvent); and (iii) explicitly treats the electrostatic interactions should be able to emulate all decisive features of studied systems and to predict the most important trends of their behavior. Therefore, we performed an extensive series of simulations using coarse-grained molecular dynamics (namely, the dissipative particle dynamics (DPD) [50,61,62,63,64]).

First, we investigated the behavior of charged porphyrins in aqueous buffers to correctly set the non-electrostatic porphyrin–porphyrin and porphyrin–solvent interactions. Second, we simulated the electrostatic co-assembly of copolymers with oppositely charged PE blocks. As the size of porphyrins is relatively large, successful modelling requires a number of relatively long copolymers with highly charged blocks in the simulation box, resulting in computationally demanding simulations. Third, we simulated the partitioning of porphyrins between IPEC cores of nanoparticles and the bulk solvent.

In brief, the primary goal of the paper is obtaining information on the solubilization of porphyrins into IPEC complexes. Knowledge of whether they solubilize in cores of polymeric nanoparticles and whether they associate there is extremely important for medical applications (e.g., for targeted porphyrins delivery into cells or for delayed and isotropic release of singlet oxygen from porphyrin-containing nanoparticles into tissues to be treated by photodynamic therapy [65,66,67,68,69]). As the simulated system is large, we used coarse-grained DPD, which is the only method applicable for this research. DPD enables unambiguous distinguishing of the associates from non-associated molecules but has no ambitions to discern different types of porphyrin associates for which the structure differs only a little. Individual aggregates differ mainly in the orientations (and in magnitudes) of dipole moments, which affect their photophysical properties. This paper represents a first step in the research on this topic. The next step should be a detailed study of a predefined IPEC part containing two ionic porphyrin derivatives by quantum simulations because the formation on various types of associates can be explained only by quantum chemistry arguments. However, this is the challenging task for experts in quantum simulations.

A comment on the nomenclature used: In addressing the reversible electrostatic co-assembly of PEs in polymer nanoparticles, we use the terms association and associate in line with the IUPAC recommendation. When discussing the self-assembling behavior of P${}^{+}$, which is, under the conditions of our simulation, reversible but, in the overwhelming majority of practically useful real systems, irreversible, we use the terms aggregation and aggregate in line with the literature on porphyrin-based systems.

## 2. Model and Simulation Technique

In this computer study, we use dissipative particle dynamics (DPD) with explicit electrostatics. As the principles of DPD have been described in the literature [61,70] and details on our variant are outlined in earlier papers [54,71], we do not include a DPD description here.

The basic setting of electrostatic and non-electrostatic forces obeys basic assumptions of classical theories of PE solutions. The DPD bead is characterized by soft non-electrostatic forces, the parameters of which are derived from the Flory χ-parameters [61] and by the charge *q*, which is set in line with most PE theories, q=e. In contrast to the fine, coarse-grained (e.g., beads spring) models of PE chains which commonly use the Lennard–Jones and the Coulomb potentials (both diverging at short distances), in DPD, where the non-electrostatic forces between beads are soft, the use of the Coulomb potential is prohibited because its combination with DPD forces would lead to nonphysical catastrophic consequences, and hence, various computational tricks have to be employed. We use the most common approach, which consists in the use of potentials describing the interaction between the exponentially delocalized electric charges. In our earlier papers, we devoted great care to the parametrization of both non-electrostatic and electrostatic forces [49,50,51,52,54,71]. In contrast to the papers of other authors, we used a smaller charge delocalization (smearing constant λ=0.2), which secured that the whole charge *e* was located in the volume of one DPD bead.

As DPD safely discerns P${}^{+}$ monomers from their aggregates but cannot differentiate between different types of aggregates that have very similar structures (indiscernible at the DPD coarse-grained level) and differ mainly in the orientation of dipole moments, we used a simple criterion for the identification of aggregates based on the close approach of beads from different P${}^{+}$ molecules (comparable with the length of bonds in polymer chains). We did not analyze the structure of porphyrin associates because we believed that the analysis of aggregates would exceed the justified interpretation of coarse-grained data.

### Parameter Setting

**Figure 1:**
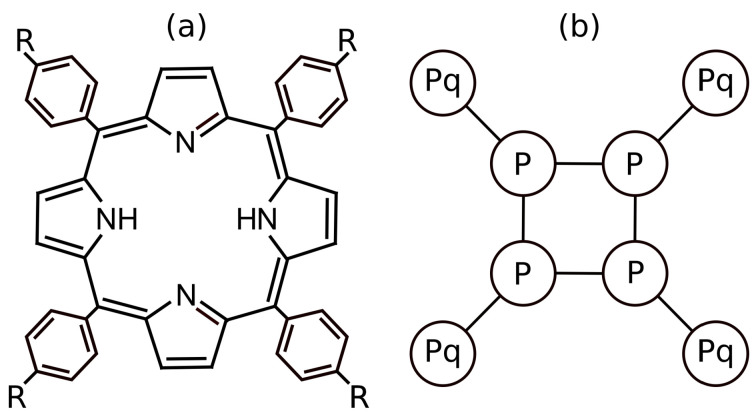
(**a**) Molecular structure of *p*-substituted porphyrin and (**b**) coarse-grained model of the porphyrin, where the square formed by P circles corresponds to the porphyrin aromatic ring and Pq is the charged groups.

In this part, we elucidated the calibration of non-electrostatic interactions. As already explained, the rigid aromatic ring containing 22 fully delocalized π electrons is strongly hydrophobic, which restricts the solubility of porphyrins in water. Porphyrins are further apt to stack all types of π–π, i.e., to “sandwich”, “T-shaped”, and “parallel displaced” interactions, and therefore, they aggregate in aqueous media [16]. The aggregation is usually promoted by interactions with solid surfaces, particularly hydrophobic ones, and with various inorganic scaffolds and large rigid molecules [72,73,74]. Ionic porphyrins bear several hydrophobic benzene or toluene rings, p-substituted by –SO${}_{3}^{-}$, pyridinium, –N^+^(CH_3_)_3_, etc. External aromatic rings are covalently attached to C atoms interconnecting the pyrrole units. (Figure 1a). Our coarse-grained porphyrin model consists of a rigid square of 4 DPD beads modelling the hydrophobic aromatic ring, each bearing one positively charged DPD bead; the whole structure is kept planar (Figure 1b).

The presence of charged groups promotes solubility in aqueous buffers and the repulsion between even charges hinders aggregation. However, the strong π–π stacking in aqueous media successfully competes with electrostatic repulsion and appreciable fractions of dimers, and other aggregates coexist in equilibrium with monomer molecules depending on the conditions (number and chemical nature of charged groups, concentration, ionic strength, temperature, etc.). Experimental studies [23,75] show that dilute low ionic strength solutions of positively charged tolyl-pyridinium-substituted porphyrins contain fairly high fractions of monomers but nonnegligible fractions of dimers, and higher aggregates were detected at elevated concentrations and ionic strengths. The authors could not determine the absorption coefficients of individual species, which prevented quantitative evaluation of weight fractions. They concluded that the increase in porphyrin concentration and in ionic strength promotes association, and these trends were also confirmed in later reports [76,77]. We used these observations as a guideline for the calibration of non-electrostatic forces acting between porphyrin molecules in aqueous media. In short, appropriately chosen repulsion parameters have to reproduce the association process well at a semiquantitative level. The proper calibration of repulsive forces with respect to electrostatic forces should secure dilute aqueous solutions of charged porphyrins containing high fraction of monomers in salt free solutions and considerable fractions of aggregates in solutions with elevated salt contents. We underline that, in contrast to a number of papers based on simplified DPD models [78,79,80], we use the “computationally expensive” DPD variant, which includes electrostatics and explicitly treats the effect of charges. The calculations are time-consuming, but the electrostatic interactions are described correctly by potentials acting between slightly delocalized (smeared) charges, and the complex electrostatic and entropic role of small counterions is appropriately taken into account [71,81].

Groot and Warren [61] elaborated the procedure suitable for the calibration of soft non-electrostatic forces on the basis of the dependence of compressibility of the system on density. They showed that the parameter $a_{ii}$ describing the interaction between two like particles that do not interact by specific attractive interactions is $a_{ii}=25$ (for the commonly used reference DPD density ρ=3). The $a_{ii}=25$ value applies, e.g., for the solvent–solvent interactions in simple liquids. The authors mapped the DPD results onto the Flory–Huggins systems of neutral (non-polar) polymer solutions and blends and found a simple relation between $a_{ij}$ and the Flory interaction parameter χ [61]:

(1) $$a_{ij}=25+3.27\chi .$$

Even though the χ-parameter has been introduced by Flory in polymer science [82], the approach was inspired by analogous concepts based on the comparison of cross interactions $w_{ij}$ with the average of homo-interactions, $\Delta w=w_{ij}-\left(w_{ii}+w_{ij}\right)/2$, which is common in physical chemistry of low-molar-mass mixtures [83]. Therefore, the considerations based on χ (note that Δw and χ are proportional to each other) can be safely employed in porphyrin-containing systems. Briefly, the values 25, 26.6, and 39.7 correspond to χ=0 (good athermal solvent), 0.5 (θ-solvent), and 4.5 (poor solvent or non-solvent), respectively. Specific attractive interactions are modelled by $a_{ij}$ values lower than 25.

**Figure 2:**
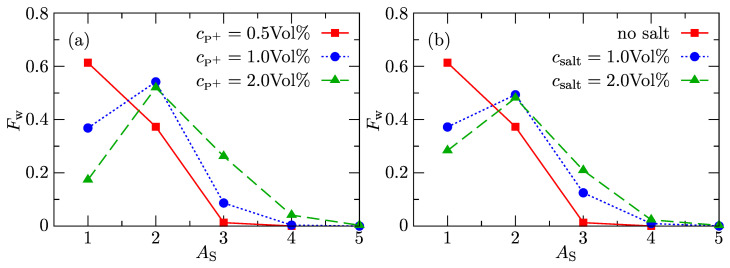
Weight distribution function of aggregation numbers $F_{w}\left(A_{S}\right)$ for (**a**) different amounts of porphyrins without added salt and (**b**) different amounts of added salt at porphyrin concentration $c_{P^{+}}=0.5$ Vol%.

To obtain the $a_{ij}$ interaction parameters, we performed a series of simulations, maintaining the standard value $a_{ij}=25$ between like particles, i.e., for solvent–solvent (S–S), counterion–counterion (CI–CI), and solvent–counterion (S–CI) interactions, for mutual interaction of external aromatic beads (Pq–Pq) and for interaction of porphyrin ring beads with external aromatic bead (P–Pq) and varying the interaction between the pairs of porphyrin ring beads (PP). The comparison with experimental data on aqueous solutions of charged porphyrins [23] yielded reasonable estimates of interaction parameters for the remaining interactions: $a_{\mathrm{PP}}=18$ and $a_{\mathrm{PS}}=a_{\mathrm{Pq}-S}=39$. The weight-average distribution functions, $F_{w}\left(A_{S}\right)$, depicting the fractions of monomers and of different aggregates in salt-free aqueous solutions with increasing porphyrin concentration are shown in Figure 2a, and the effect of added salt on the association process is depicted in Figure 2b. The changes in shapes of $F_{w}\left(A_{S}\right)$ show a substantial increase in fractions of dimers and higher aggregates with both porphyrin concentration and ionic strength. This reasonable reproduction of experimental trends at the semiquantitative level provides evidence that the parameters of repulsive forces (P–P, P–S, and Pq–S) have been appropriately set.

We studied the co-assembling systems of block copolymers A${}_{10}$B${}_{25}$, where A denotes the PE blocks (both positively and negatively charged) and B is the beads of the neutral water-soluble polymer block (NWP). A copolymer with a longer NWP block than the PE block secures better stability of co-assembled nanoparticles [40], but we chose it for a different reason. Some of the coauthors of this communication planned systematic experimental studies on the solubilization of porphyrins into electrostatically co-assembled polymeric nanoparticles, and we adjusted the simulation model to the copolymers they intend to use.

As already explained, the backbone of common PEs is hydrophobic and PEs dissolve in water thanks to the presence of charged groups incorporated in or attached to the chains. The non-ionized poly(2-vinylpyridine) and poly(4-vinylpyridine), which represent suitable PE candidates for the design of porphyrin-containing systems, are strongly hydrophobic and do not dissolve in neutral and alkaline solutions. The oppositely charged counterparts (e.g., sulfonated polystyrene) also contain strongly hydrophobic backbones. Based on our earlier studies, in which we successfully emulated the behavior of self- and co-assembling PE systems [52,54,71], and with respect to the length of PE block, we selected a medium-high repulsion value $a_{AS}=35$. According to our earlier results, this value represents the molecular solubility limit (onset of micellization) for a neutral A${}_{5}$B${}_{5}$ copolymer with a well-soluble block B. However, the studies showed that compact core-shell associates with well-segregated IPEC cores form in A${}_{5}^{+}$B${}_{5}$ and A${}_{5}^{-}$B${}_{5}$ mixtures when $a_{AS}=35$. Most of commonly used neutral water-soluble polymers, i.e., poly(ethylene oxide) (PEO), poly(*N*-isopropyl acrylamide) (PNIPAM), and polyoxazolins (PEOX), readily dissolve in aqueous media, but their solubility is limited and depends strongly on temperature and ionic strength. Therefore, we did not use $a_{BS}=25$ but rather a slightly higher value of 26. Because the hydrophobic PE backbone is usually incompatible with the polar, water-soluble block, we used $a_{AB}=35$. Lastly, we assumed that the non-electrostatic interactions of counterions with other components are the same as those of solvent molecules with the exception of A–CI interaction, which was set to $a_{A-\mathrm{CI}}=27$ to account for important attractive short-range interaction of ions with pendant aromatic rings (pyridine or benzene) on PE chains [84,85,86].

All interaction parameters are listed in Table 1.

## 3. Results and Discussion

### 3.1. Electrostatically Stabilized Core-Shell Nanoparticles with IPEC Cores without Solubilized Porphyrin

We studied the electrostatic co-assembly of oppositely charged block polyelectrolytes by coarse-grained simulations for almost ten years [49,51,52,54,71]. Our extensive studies of both stoichiometric systems with matched positive and negative charges on PE blocks and of nonstoichiometric systems with excess of either positive or negative charges (i) confirmed already known facts, e.g., the importance of the entropy of counterions, which is the driving force of the assembling processes; (ii) revealed the conditions necessary for the formation of nanoparticles with well-segregated domains; and (iii) indicated new important behavioral trends. We underline that the formation of well-defined core-shell particles requires some hydrophobicity of IPEC-forming PE chains (moderate to significant) and their (at least partial) incompatibility with the water-soluble shell-forming chains. Otherwise, highly irregular gel-like particles (very disperse in size) with intermixed PE and water-soluble blocks form in mixtures of oppositely charged PE-NWPs.

**Figure 3:**
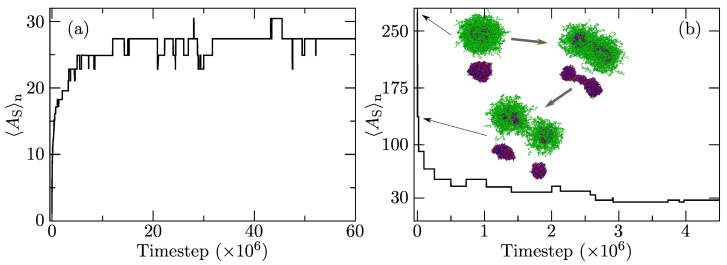
Time evolution of the number-averaged association number, ${\langle A_{S}\rangle }_{n}$, from (**a**) a fully dissolved state and (**b**) a pre-aggregated state with snapshots of the breakup of the initial associate. In the snapshots, the red and blue beads represent the core-forming A${}^{+}$ and A${}^{-}$ blocks, respectively, and the green beads represent the shell-forming B blocks (the upper images are whole associates, while the lower ones show their interpolyelectrolyte complex (IPEC) cores).

In this study, we performed simulations of a system containing 137 A${}_{10}^{+}$B${}_{25}$ and 137 A${}_{10}^{-}$B${}_{25}$ copolymer chains without porphyrins starting from two different initial conditions: (i) randomly dispersed copolymer chains and (ii) fully aggregated chains (one large core-shell aggregate). The evolution of the ensemble-average association numbers, ${\langle A_{S}\rangle }_{n}$, for the system with $a_{AS}=35$ starting from the random initial state is shown in Figure 3a. Initially, the formation of small core-shell aggregates with IPEC cores proceeds very fast, with ${\langle A_{S}\rangle }_{n}$ increasing within the first $10^{6}$ timesteps up to 18; then, the increase slows down but continues up to 28 during the next $1\times 10^{7}$ steps. Then, ${\langle A_{S}\rangle }_{n}$ levels off, and the association process seems to reach an equilibrium. The slowdown of the equilibration in later times is partly due to the natural deceleration of relaxation processes as the system approaches equilibrium, but the main reason is purely mechanistic. As soon as the critical size of compact IPEC domains is reached, both the liberation and incorporation of PE chains from/into them become difficult. Steric hindrances due to a high density of the core and high energy barrier of the passage of incompatible A block through the shell formed by B blocks considerably slow the exchange of chains between the associates.

Figure 3b shows the evolution of ${\langle A_{S}\rangle }_{n}$ from the pre-aggregated state constructed as one large associate. The initial decomposition of the predefined associate is very fast, with ${\langle A_{S}\rangle }_{n}$ dropping from 274 to ca 90 during the first $2\times 10^{4}$ timesteps. This slightly surprising observation can be rationalized as follows: Even though the associate was constructed as a core-shell particle and enthalpy requirements for its stability were fulfilled, ${\langle A_{S}\rangle }_{n}$ was much higher than the equilibrium one. The IPEC core was large, and the conformations of the core-forming PE chains were far from optimal ones, which restricts the entropy contribution to the Gibbs function. As shown in the snapshots in Figure 3b, the large spherical core deforms, passes through a dumbbell shape, and splits into two roughly spherical nanoparticles within the first $10^{4}$ timesteps. Within the next $10^{4}$ steps, the larger particle splits again. As the average size approaches the equilibrium size, the process decelerates considerably, which can be rationalized as follows: In this region of association numbers, the splitting of associates leads to a drop in $A_{S}$ below the equilibrium average value, and therefore, the splitting stops and is replaced by sterically and energetically less favorable release of individual chains (or pairs of oppositely charged chains) from the associates, which in the bulk solvent start to form a new associate. The final equilibration step is much slower than the initial one, but the association number of resulting micelles, ${\langle A_{S}\rangle }_{n}$, approaches that obtained when the simulation starts a random configuration of chains.

**Figure 4:**
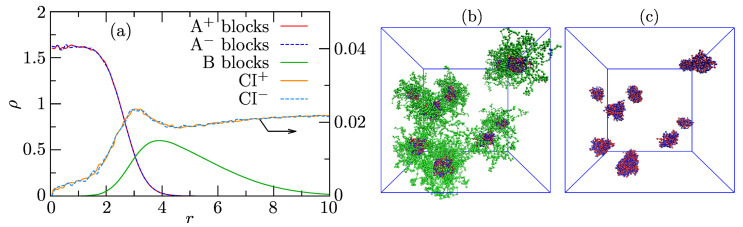
(**a**) Radial density profiles (RDPs), ρ, of the associate with $A_{S}=30$ and (**b**,**c**) typical snapshot of the simulation box containing associates and their IPEC cores, respectively, where red and blue beads represent the core-forming A${}^{+}$ and A${}^{-}$ blocks, respectively, and the green beads represent the shell-forming B blocks.

Ensemble- and angularly averaged radial density profiles (RDPs) of components in highly populated associates with $A_{S}=30$ are depicted in Figure 4a. The shapes of the A${}^{+/-}$ and B profiles unambiguously prove the structure of spherical core-shell associates with strongly segregated IPEC cores and B shells. The segment densities of oppositely charged PEs in the core are high, and the charges are mutually compensated in the whole core region. The density of shell-forming B blocks is appreciably lower and decreases towards the shell periphery. The increase in B density in the interfacial region of normalized distances, *r*, from 3 to 5 and the apparent overlap of B and A${}^{+/-}$ profiles between 2 and 4 suggesting some intermixing of A${}^{+/-}$ and B beads are mainly artifacts of the angular averaging of instantaneous structures, the nonnegligible fraction of which appreciably deviates from spherical symmetry [71]. The intermixing of PE blocks with B is in fact very small. Despite the fairly high compatibility of counterions with both PE blocks ($a_{A-\mathrm{CI}}=27$), the concentration of small ions inside the core is low. This observation reflects the fact that electric charges on PE chains are mutually compensated, and the core (as a whole) is neutral. However, the main reason is IPEC compactness, which restricts the mobility of small ions in the cores, lowering their translational entropy. The concentration of ions slightly increases in the core-shell region because the core surface, though on average neutral, contains individual A${}^{+}$ and A${}^{-}$ beads, and the small ions feel individual charges at distances comparable to their size. Then, the concentration of ions slightly drops and remains almost constant in shells and in the bulk solution. Note that the scales on the left-hand side and right-hand side axes for polymer beads and counterions, respectively, differ by two orders of magnitude. Figure 4b shows a typical snapshot of a simulation box containing a mixture of associates, and Figure 4c shows a snapshot of their IPEC cores only.

### 3.2. Solubilization of Cationic Porphyrins into IPEC Cores

**Figure 5:**
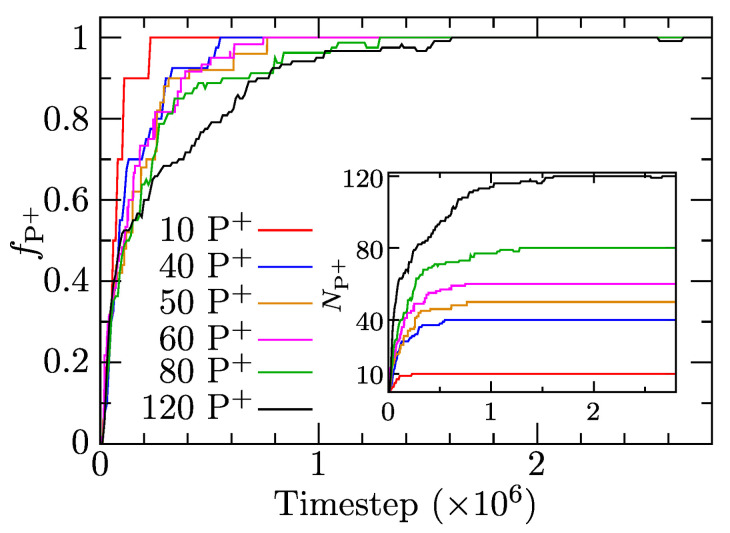
The fraction of porphyrins solubilized in the associates, $f_{P^{+}}$, as a function of simulation time for all systems with added porphyrin. Insert: the number of solubilized porphyrins, $N_{P^{+}}$, as a function of time.

We simulated the solubilization of ionic porphyrins in IPEC cores of associates prepared both by association from a random state and by dissociation of the predefined non-equilibrium associate. Dissolved porphyrin molecules were inserted into a bulk solvent at random without touching the associates. Because the behavior and structural characteristics are the same for both cases, we present only the results for the former system. We investigated the solubilization of various amounts of charged porphyrins (from 10 to 120 P${}^{+}$ molecules) into IPEC cores in a system composed of 137 A${}_{10}^{+}$B${}_{25}$ and 137 A${}_{10}^{-}$B${}_{25}$ copolymer chains with $a_{AS}=35$. Figure 5 depicts the rates of solubilization as fractions of solubilized P${}^{+}$, $f_{P^{+}}$ and number of solubilized P${}^{+}$, $N_{P^{+}}$ as functions of simulation time, showing that P${}^{+}$ solubilizes quantitatively and quickly in IPEC cores in all cases. In the case of low loading (10 P${}^{+}$ molecules added), the incorporation of all molecules is complete in $2.5\times 10^{5}$ steps. The initial rate is very fast in all cases, and 50% uptake is always reached in under $2\times 10^{5}$ timesteps. Further uptake slows down depending on the number of added P${}^{+}$ molecules, and the complete solubilization of 120 P${}^{+}$ requires more than 6 times longer than that of 10 P${}^{+}$, but it is still quite fast ($1.6\times 10^{6}$ timesteps) compared with the simulation length ($10\times 10^{6}$ timesteps), which means that the average characteristics presented later are sufficiently equilibrated.

**Figure 6:**
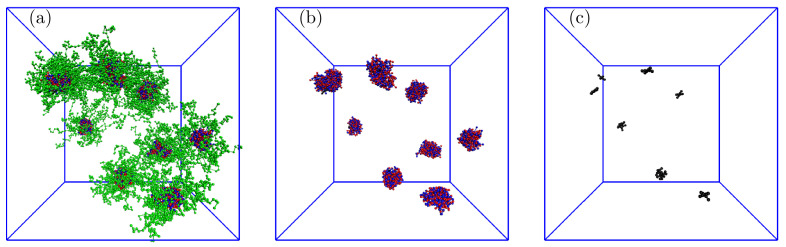
Typical snapshot of the simulation box for the system with 10 P${}^{+}$ showing (**a**) whole associates, (**b**) IPEC cores with P${}^{+}$, and (**c**) P${}^{+}$ only: the red and blue balls represent positively and negatively charged A blocks, respectively; the green balls are neutral B blocks, and the black balls are P${}^{+}$ beads.

We studied the systems with solubilized porphyrins in detail, starting from the lowest number of 10 P${}^{+}$. As the number of P${}^{+}$ in the simulation box was comparable to the average number of PE aggregates, we expected that the overwhelming majority of polymer associates contained one solubilized P${}^{+}$, but the simulation results were slightly different. A typical snapshot of the simulation box in Figure 6 (Figure 6a–c show whole associates, their cores, and P${}^{+}$ only, respectively) shows that the associates contain from 0 to 3 P${}^{+}$ molecules; specifically, two associates contain no P${}^{+}$, five associates contain 1 P${}^{+}$, one associate contains 2 P${}^{+}$, and one associate contains 3 P${}^{+}$. Note that on the left side of the snapshot are two associates positioned one behind the other. As the simulation run used for the data evaluation on equilibrated systems was 100 times longer than that necessary for a complete porphyrin uptake into associates, the observed nonuniform distribution of porphyrins among associates, reflecting the porphyrin proclivity towards aggregation in the IPEC cores, described without any doubt the true equilibrium behavior of the low-loaded system.

**Figure 7:**
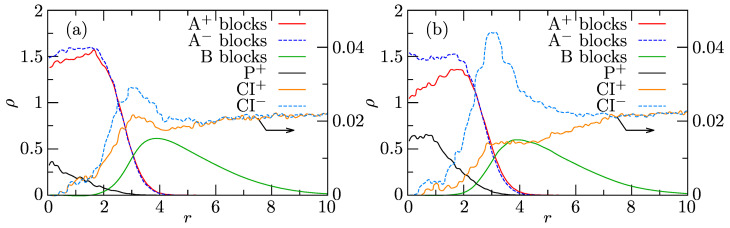
Radial density profiles, ρ, of the associates from the system with 10 P${}^{+}$ composed of 15 PE${}^{+}$ and 15 PE${}^{-}$ with (**a**) 1 P${}^{+}$ or (**b**) 3 P${}^{+}$ solubilized in its IPEC core.

Figure 7a,b show the radial density profiles of components in associates composed of 15 PE${}^{+}$, 15 PE${}^{-}$, and 1P${}^{+}$ and of 15 PE${}^{+}$, 15 PE${}^{-}$, and 3 P${}^{+}$, respectively. The shapes of RDPs of individual components confirm that the associates with solubilized porphyrins retain the core-shell structure and that P${}^{+}$ solubilizes in their cores. Porphyrin is located preferentially in the central part of the core, where it partially replaces the PE${}^{+}$ chains. In the latter case with 3 P${}^{+}$, the difference in concentrations of PE${}^{+}$ and PE${}^{-}$ beads in the central core region containing P${}^{+}$ rings is clearly seen. Before discussing higher P${}^{+}$ loadings, we underline that the formation of P${}^{+}$ aggregates in the IPEC cores starts at very low loadings thanks to the strong aggregation tendency of conjugated P${}^{+}$ rings and their favorable interactions with the IPEC-forming PEs (both electrostatic and non-electrostatic).

**Figure 8:**
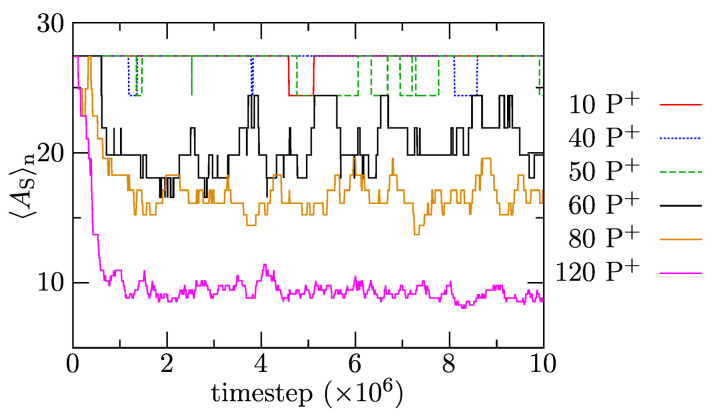
Average association number, ${\langle A_{S}\rangle }_{n}$, as a function of simulation time for systems with added P${}^{+}$.

In the next part, we focus on the effect of increasing amounts of P${}^{+}$ solubilized in IPEC cores on the composition and structure of associates. Figure 8 depicts the time evolution of the number-averaged association numbers of polymer components (including the single chains with $A_{S}=1$ but excluding P${}^{+}$) to show how the increase in P${}^{+}$ amounts in the mixture affects the release of positively charged PEs that associates into bulk solution. The results show that, in systems with up to 50 added P${}^{+}$ molecules, the solubilized porphyrins do not trigger the release of positively charged copolymer chains into the bulk solvent, and ${\langle A_{S}\rangle }_{n}$ remains constant upon solubilization. However, increased loadings (the addition of more than 60 P${}^{+}$ into the mixture) provoke a fairly fast release of PE${}^{+}$ copolymers witnessed in a significant decrease in ${\langle A_{S}\rangle }_{n}$. As expected, the driving force for the solubilization increases with the P${}^{+}$ content, which translates to a larger decrease and smaller fluctuations in ${\langle A_{S}\rangle }_{n}$ with time in the system with 120 than 80 P${}^{+}$. In agreement with the previously presented data, the P${}^{+}$ solubilization-induced release of PE${}^{+}$ copolymers proceeds fast and is complete in ca. $10^{6}$ timesteps. In summary, the results indicate that a relatively sudden and intuitively unexpected change in chain reorganization occurs between 50 and 60 added P${}^{+}$. However, the existence of a certain “dead P${}^{+}$ concentration region” is understandable. Both P${}^{+}$ and PE copolymers are relatively soluble and multiply charged, but the latter bears more charges and their molar mass is ca. 4.4 times larger. The replacement of one PE chain requires the equivalent of 2.5 P${}^{+}$, and its release into bulk solvent (compensated by P${}^{+}$ uptake) is entropically unfavorable. This, we believe, hinders the release of PE copolymers at low P${}^{+}$ loadings.

**Figure 9:**
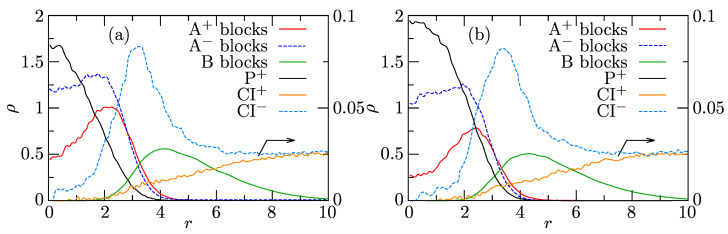
Radial density profiles, ρ, of associates from the system with 120 P${}^{+}$ composed on average of (**a**) 14.6 PE${}^{+}$, 15 PE${}^{-}$, and 9.0 P${}^{+}$ and (**b**) 12.1 PE${}^{+}$, 15 PE${}^{-}$, and 15.2 P${}^{+}$.

**Figure 10:**
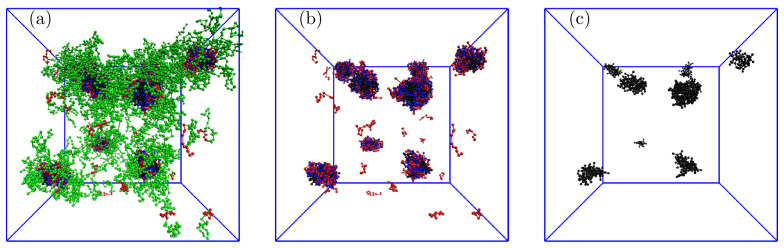
Typical snapshot of the simulation box for the system with 120 P${}^{+}$ showing (**a**) whole associates, (**b**) IPEC cores with P${}^{+}$, and (**c**) P${}^{+}$ only: the red and blue balls represent positively and negatively charged A blocks, respectively; the green balls are neutral B blocks, and the black balls are P${}^{+}$ beads.

The main accent was put on the study of high P${}^{+}$ loadings in the IPEC cores, particularly on the mixture containing comparable numbers of individual components, i.e., 120 P${}^{+}$, 137 A${}_{10}^{+}$B${}_{25}$, and 137 A${}_{10}^{-}$B${}_{25}$. Figure 9a,b show the effect of different P${}^{+}$ uptakes on the structure of the IPEC cores. These two associates contain on average either 14.6 PE${}^{+}$, 15 PE${}^{-}$, and 9.0 P${}^{+}$ or 12.1 PE${}^{+}$, 15 PE${}^{-}$, and 15.2 P${}^{+}$. The analysis of their structure confirms the conclusions stated earlier: the solubilized P${}^{+}$ molecules accumulate close to the IPEC center, replace the positively charged PE blocks, and electrostatically crosslink the oppositely charged PE blocks. Figure 10 presents a snapshot of typical associates (a), their IPEC cores (b), and P${}^{+}$ molecules (mostly the aggregates formed within the IPEC cores).

An important message from the study can be formulated as follows: Even though the multiply charged P${}^{+}$ are more soluble in aqueous media than their neutral counterparts, they accumulate in IPEC cores (replacing some positively charged PE blocks and electrostatically crosslinking the negatively charged PE blocks), thus contributing to the stability of the nanoparticles. P${}^{+}$ solubilization is promoted (i) by multiple electrostatic interactions in combination with the entropically favorable release of small counterions into bulk solvent and (ii) by attractive nonelectrostatic interactions of the aromatic P${}^{+}$ ring with the PE backbone, particularly in the case of fairly hydrophobic PEs bearing pendant aromatic substituents, such as poly(4-vinylpyridine) or sulfonated polystyrene. The analysis of IPECs reveals that cores with solubilized P${}^{+}$ are in contrast to the parent nanoparticles positively charged and that the charge, which appears mainly at the core-shell interface, is compensated by increased concentrations of small ions at the core-shell interface.

As the distribution function of associated PE copolymer chains in the polymer assembly without added P${}^{+}$ is fairly broad, we analyzed both the general trends of the behavior of the most populated associates with ${\langle A_{S}\rangle }_{n}$ and the effects in associates with $A_{S}$ < ${\langle A_{S}\rangle }_{n}$ and $A_{S}$ > ${\langle A_{S}\rangle }_{n}$, where ${\langle A_{S}\rangle }_{n}$ represents the number-averaged association without added P${}^{+}$. Depending on the fraction of porphyrins in the mixture and on the size of copolymer associates, the positively charged PEs, which are expelled from the central part of the core, either partially accumulate on the core-shell interface (which becomes slightly charged) or escape into bulk solution. In small associates with $A_{S}<{\langle A_{S}\rangle }_{n}$, they preferably concentrate on the core-shell interface, thus increasing their overall mass. In large associates with $A_{S}>{\langle A_{S}\rangle }_{n}$, the positively charged copolymers are released mainly into the bulk solvent, which leads to a decrease in the molar masses and sizes towards the ensemble-averaged values. While the basic trends of the behavior are well-pronounced, the system is dynamic: Both types of copolymer chains and P${}^{+}$ exchange relatively fast between associates, and $A_{S}$ of individual associates as well as the numbers of P${}^{+}$ molecules solubilized therein strongly fluctuate with time, which is not surprising because all components are relatively soluble in aqueous media.

## 4. Summary and Conclusions

Urged by the surprising lack of experimental as well as simulation data on the solubilization of positively charged porphyrin derivatives in IPEC cores of nanoparticles formed by electrostatic co-assembly in stoichiometric mixtures of copolymers containing a neutral water-soluble block and a hydrophobic PE block (either positively or negatively charged), we performed an extensive series of coarse-grained DPD simulations with explicit electrostatics aimed at their co-assembly and P${}^{+}$ solubilization.

Based on these results (the large collection of typical snapshots, distribution functions, radial density profiles, and auxiliary plots of evaluated characteristics considerably exceeds the size of reasonable Supplementary Materials, so the data are available upon request), we can conclude the following: (i) Positively charged P${}^{+}$ easily solubilizes in IPEC cores of electrostatically co-assembled nanoparticles formed in stoichiometric mixtures of PE${}^{+}$ and PE${}^{-}$ components. (ii) They partly replace the evenly (i.e., positively) charged PEs. (iii) Being multiply charged, they electrostatically crosslink the negatively charged PE blocks. (iv) As the large P${}^{+}$ rings with 22 fully delocalized π electrons are apt to aggregate, P${}^{+}$ aggregates are formed in IPEC cores and the aggregation starts at very low loadings. (v) Equilibrium systems contain a relatively broad distribution of associates differing in numbers of associated components and in numbers of solubilized porphyrins. (vi) In associates containing only a few P${}^{+}$, the IPEC cores contain comparable numbers of PE${}^{+}$ and PE${}^{-}$, while in the highly P${}^{+}$-loaded associates, the numbers of negatively charged PEs significantly exceed those of positively charged PEs. (vii) However, in all systems and particularly in those prepared by mixing with high amounts of P${}^{+}$, all associates differing in molar mass and composition (i.e., those with both high and low P${}^{+}$ contents), are appreciably positively charged and the charge is compensated by small counterions concentrating at the core-shell interface.

As already mentioned, the used coarse-grained approach safely distinguishes P${}^{+}$ monomers from aggregates but does not have ambitions to discern between different types of porphyrin aggregates for which the structure differs only a little. Our DPD simulations yield two important findings and demark the direction for future research: (i) ionic porphyrins solubilize in IPEC domains and (ii) form aggregates in those domains. The question of which type of aggregate prevails in a given IPEC medium is a challenging topic for experts in quantum simulations.

Last but not least, our study aimed at realistic (readily applicable) PE–porphyrin systems, in which the PE charges are located at pendant aromatic rings (e.g., poly(2-or 4-vinylpyridine) or *p*-sulfonated polystyrene) and the short-range attractive forces between ions and delocalized π electrons in aromatic rings play an important role. Therefore, we used a low value of the interaction parameter $a_{A-\mathrm{CI}}$, modelling the ions as fairly compatible with the IPEC complex. In spite of that, the study shows that the increase in entropy upon the escape of small monovalent ions from dense IPEC cores into bulk solvent overbalances all attractive (short-range non-electrostatic and long-range electrostatic) interactions and the concentrations of ions in the core is very low in all studied systems.

## Figures and Tables

**Table 1:** Interaction parameters.

	$a_{\mathrm{ij}}$					
	A${}^{+/-}$	B	P	Pq${}^{+}$	CI${}^{+/-}$	S
A${}^{+/-}$	25					
B	35	25				
P	25	35	18			
Pq${}^{+}$	25	35	25	25		
CI${}^{+/-}$	27	26	39	39	25	
S	35	26	39	39	25	25

## Data Availability

The data presented in this study are available on request from the corresponding author.
